# Annotations

**Published:** 1890-06-07

**Authors:** 


### Annotations.

**Unsectarian Hospitals.**

What hospitals are unsectarian? Most of them, both in London and the country. This appears a very broad statement to make, and no doubt many good people will call it in question. But a little saving common sense may not unreasonably be looked for, even at the hands of those who are dealing with ecclesiastical and political questions. A hospital is not to be branded with sectarianism merely because the chairman of its committee asks a matron or a nurse what religious denomination she delongs to. It ought not to be forgotten that the Church of England has long occupied a position of supremacy, and that for many years a large proportion of the wealth, culture, and public influence of the country has been on her side. If, under these circumstances, Churchmen have sometimes seemed inclined to take the leadership with a little too much decision, it is not at all surprising, nor should their action be judged too harshly. There is a good deal of human nature even in Churchmen. On the other hand, it must be remembered that Nonconformists have their profound and firmly-held convictions. Those convictions have sometimes led their holders to a certain amount of loss and injury of various kinds. On that account they are often held all the more firmly. For a man is very apt to think, and not unnaturally, that a conviction for which he has suffered must needs be both intelligent and honest. We are anxious, therefore, that Churchmen should forget their assured position and Nonconformists their real and supposed grievances in dealing with hospital questions. We cannot but think that many Nonconformists are altogether too sensitive on the subject of their Nonconformity. They do not sufficiently bear in mind that there is always a certain amount of honour attaching to conscientious conviction. They seem to forget also that it is possible to carry dissent of any kind to lengths which good sense will not warrant. Dissenters think that Churchmen should be kind and brotherly towards them, and so do we. But whatever obligation rests upon Churchmen to be kind and brotherly towards Dissenters lies with equal weight upon Dissenters, not only to reciprocate the feeling, but even to initiate it. It was the Master of both Churchmen and Dissenters who said, "Blessed are the peacemakers;" and who added to it this striking reason, "For theirs is the kingdom of heaven." The writer has been familiar for many years with both Anglican and Nonconforming pulpits, and he never remembers to have heard this text preached from, even on a single occasion. Yet the kingdom of heaven is the very thing that every church persistently claims to represent, and the very thing that every church professes to exist for no other purpose but to represent. What a striking circumstance it is that the first condition of the possession of the kingdom of heaven is hardly ever alluded to by those who claim to be custodians and teachers of the mysteries of that kingdom! We have often urged that Hospital Sunday furnishes an occasion for the laying down of the weapons of controversy, and the shaking of hands. Blessed is that man or that organization which is readiest to disarm and promptest to seek the enemy's camp with a flag of truce.

**Count Mattei, the Cancer Curer.**

Does Count Mattei really cure cancer? Lady Paget, in the *National Review*, says he does, and says it with a great deal of circumstantiality and fulness of detail. It ought to be quite possible for the Italian doctors, at any rate, to prove whether the Count cures cancer or not; and if he does, it is certainly their duty to recognize the value of his treatment, and to secure his secret, if secret there be, for the use of mankind at large. For ourselves, we frankly confess our entire disbelief in the Count's power to cure. But we as frankly affirm, not only our readiness, but our eagerness, to be convinced by actual facts. Lady Paget tells a marvellous story of the discovery of the life-giving plant by a mangy dog. She also unfolds the wonders of what she or the Count, or both of them, call "blue electricity." She gives an account of an American boy of eighteen who, when brought to the great man, was a huge, unshapely, and scarcely human monster, and who appeared to be the victim at one and the same time both of elephantiasis and leprosy. He looked nearer seventy than seventeen years of age. After treatment by the Count all his hideous grossness disappeared, and he had the look of a thin and elegant youth of sixteen. The late Pope Pius IX., it is said, ordered to be given up to the Count a part of the hospital at Sta. Teresa twenty years ago, and a large number of cures are said to have been then effected. It is stated that a fully qualified medical man in London, whose name we do not intend to give, is practising the Count's treatment. No doubt many unfortunate sufferers have read or heard of Lady Paget's article, and are probably now in a state of the keenest and most distressing suspense. Has there really at last been found a cure for their terrible malady? The question is agonising! They do not trouble themselves whether the cure is to be effected by orthodox or unorthodox means. And the grimmest defender of orthodoxy will not dare to blame them if the "cure" be genuine. But we have expressed our conviction that it is not. If it be, then the London medical man who practises the treatment ought at once to make it known to all his medical brethren, and to the country at large. If he does not, at his door should lie the guilt of the untimely death of all those who may hereafter be the victims of cancer in this country. The very fact that no attempt is made to bring the subject before the medical profession, when such a course, if the cure were proved to be genuine, would raise the discoverer to the highest pinnacle of honour, and load him with wealth—this fact alone seems conclusive against the *bona fides* both of Count Mattei and his London medical follower.

**Merry May at Paddington.**

Two Thousand Five Hundred boys and girls from the North-West Districts of London assembled at the Paddington Recreation Grounds on Whit-Monday for a day of old-fashioned sports. About 17,000 older people also came to look on. The day was uncommonly fine, and the sports brilliantly successful; and it is safe to say that, of the whole 17,000 spectators, there was hardly one that did not wish to be young again in order to join in the fray. For the 150 yards race for boys under eleven years of age, there were no fewer than 559 entries; 233 girls, under eleven, entered for the 100 yards foot-race; and 271 girls, over eleven, for a similar race. There were skipping-rope races, egg and spoon races, hoop, sack, and obstacle races of every conceivable kind. No doubt Whit-Monday proved to many of the children the "maddest, merriest day of all the glad new year." The Earl of Meath, Sir George Harriss, Mr. John Aird, M.P., Mr. H. W. Lawson, M.P., and others had generously given many of the prizes. What pleases us, from a health point of view, is the old-fashioned, strenuous, and athletic character of the sports, and the thorough appreciation of them by the London boys and girls who took part, and by their parents, uncles, aunts, and grandmothers, who went to look on. London might almost be turned into a village again if only we had a "green" big enough, and a Maypole sufficiently tall. It is a pity that such delightful sports and pastimes cannot take place weekly, or, at least, monthly, instead of annually. Nothing would better serve the purposes of health and recreation for the tens of thousands of boys and girls, whose only playground is the crowded and, too often almost airless, street. In London people do not play half enough; those that have passed the period of youth hardly, indeed, play at all. The very children, it would seem, must, in time, also lose their regular out-door games, and become, except at rare intervals, demure and proper little creatures, to whom the art of playing is almost unknown. But we must not blind ourselves to the consequences which will certainly ensue, when sports and pastimes shall have entirely gone out of fashion. England will no longer be the England either of our forefathers or of our own ideals. It certainly will not be a better England, nor a happier. Out-door life is one of the fundamental and indispensable conditions of health and vigour; and out-door sports and pastimes of the more strenuous kinds have been chiefly conducive in making the Englishman what he is, as a warrior, as a colonist, and as a practical leader among the races of men. The population of the country is gathering more and more into the large towns, and rural sports are thereby rendered more and more difficult. The best Englishmen, and all, indeed, who wish to preserve the national character, should make it an important part of their public business to develope the national liking for athletic sports, and to supply places and opportunities for their exercise. Our national character has raised us to the front rank among imperial races; and any failure to reach the intellectual and physical level of our forefathers, will expose us to the risk of having to take a second or third place.

